# Whole-genome sequencing and in vitro characterization of two lactic acid bacteria isolates with potent antimicrobial activity from fermented vegetables

**DOI:** 10.1186/s12866-026-05132-2

**Published:** 2026-05-18

**Authors:** Lianci Peng, Yiming Wang, Shichao Xu, Zhouyuan Wang, Juan Li, Xuefeng Cao, Yunxia Li, Rendong Fang, Xingxing Dong, Zhiwei Li

**Affiliations:** 1 Joint International Research Laboratory of Animal Health and Animal Food Safety, College of Veterinary Medicine, Southwest University, Chongqing, 400715 China; 2 Kunming Hemeihua Feed Limited Company, Kunming, 652100 China; 3 National R&D Center for Serich Agricultural Products Processing, School of Modern Industry for Selenium Science and Engineering, Wuhan Polytechnic University, Wuhan, 430023 China

**Keywords:** Lactic acid bacteria, Probiotic properties, Antimicrobial activity, Antioxidative activity, Fermented vegetables

## Abstract

Lactic acid bacteria (LAB) exist in various sources including the gastrointestinal tract and fermented foods, and are considered beneficial for animal and human health. In this study, two LAB strains *Lactiplantibacillus plantarum* CLPX16 and *Weissella cibaria* CLPX19 were isolated from fermented vegetables and characterized for probiotic properties through whole-genome sequencing and in vitro functional assays. Genomic analysis revealed that although homologs of several virulence-related genes were detected in both strains, they exhibited low sequence identities and lacked classical virulence gene determinants such as toxins, secretion systems, or invasion proteins. In contrast, both strains harbored beneficial genes including bacteriocin biosynthesis clusters and secondary metabolite gene clusters associated with antimicrobial and antioxidant properties. Both strains exhibited strong tolerance to acidic conditions with high survival rates at pH 2.0, 3.0, and 4.0, as well as tolerance to bile salts. Moreover, both strains exhibited robust acid production capacity, inducing a rapid pH decline during 0–36 h of incubation. In addition, both strains exhibited notable antioxidative activity, nitrite degradation capacity, and adhesion capacity. Importantly, CLPX16 and CLPX19 exhibited potent antimicrobial activity against foodborne pathogens including *Staphylococcus aureus*, *Escherichia coli*, and *Salmonella enterica.* Notably, these strains lacked hemolytic activity and showed low risk of horizontal transfer of antibiotic resistance genes. In conclusion, CLPX16 and CLPX19 demonstrate promising probiotic potential and represent candidate strains for application in food and health industries.

**Supplementary Information:**

The online version contains supplementary material available at 10.1186/s12866-026-05132-2.

## Introduction

 Many lactic acid bacteria (LAB) strains have been classified as probiotics and are widely distributed in natural environments including the gastrointestinal tracts of humans and animals, as well as various fermented foods [1]. Fermented vegetables, as one of the most popular foods in Asia, have been an important source of LAB isolates [2]. LAB are well-recognized for their multiple beneficial effects on the host. For example, they can modulate intestinal microbiota homeostasis, enhance host immune function, improve nutrient digestibility, and exert protective effects against pathogenic microorganism colonization [2]. LAB have been widely considered safe and beneficial to animal and human health. Therefore, the isolation and identification of new LAB strains will promote the development of LAB-based application and enrich probiotic resource libraries.

Whole-genome sequencing (WGS) has emerged as a powerful and indispensable tool for the comprehensive characterization of LAB strains, providing unprecedented insights into their genetic background, potential functions, and evolutionary relationships [3, 4]. WGS enables the systematic identification of genes associated with probiotic characteristics including antioxidant activity and bacteriocin biosynthesis, as well as the screening of virulence factors and antibiotic resistance genes [5]. In addition, genomic analysis combined with in vitro and in vivo functional assays provides a more accurate and comprehensive assessment of the probiotic potential of LAB isolates, which is essential for their safety evaluation and clinical application [3–5]. This genomic-driven approach has become a mainstream strategy for the discovery and characterization of novel LAB probiotic strains in contemporary microbiological research.

Foodborne pathogenic bacteria, such as *Staphylococcus aureus* (*S. aureus*), *Escherichia coli* (*E. coli*), and *Salmonella*, cause foodborne diseases, which pose a great threat to global public health and food safety [6, 7]. Foodborne pathogens are major concerns in meat products, milk, eggs, and leftover foods, causing gastrointestinal infection and leading to diarrhea, abdominal cramps, and fever. The World Health Organization (WHO) estimates that approximately 600 million people are infected annually through consumption of contaminated food, posing substantial burdens and significant risks to society [7]. In addition, the emergence of antimicrobial resistance increases the difficulty in controlling foodborne pathogen infections [8]. LAB can produce lactic acid, acetic acid, formic acid, and other acids to reduce environmental pH, which inhibits the growth of pathogenic bacteria [9]. Furthermore, LAB also exert antimicrobial activity via the secretion of antimicrobial molecules including ethanol, fatty acids, hydrogen peroxide, and bacteriocins [10, 11]. Apart from the direct antimicrobial effect, LAB can reduce harmful substances including nitrite and oxidants during fermentation, thereby enhancing the nutritional value and safety of food. Therefore, developing LAB as probiotic agents is a promising strategy to control foodborne pathogenic infections.

In this context, the present study aimed to isolate and characterize two novel LAB strains from fermented vegetables through a combination of WGS and in vitro functional assays. We further assessed phenotypic characteristics including acid and bile salt tolerance, acid production capacity, antioxidant activity, nitrite degradation capacity, and antimicrobial activity against common foodborne pathogens. Our study provides insights into the potential application of these strains as probiotics against pathogenic infections.

## Materials and methods

### Bacterial strains and culture conditions

LAB strains CLPX16 and CLPX19 were isolated from fermented radish collected in Binzhou City, Shandong Province and Xinxiang City, Henan Province, respectively. Strains were cultured on Man-Rogosa-Sharpe (MRS; Oxoid Inc., United States) agar plates or in MRS broth at 37 °C. Stocks were stored at -80 °C in MRS broth containing 25% glycerol until use.

Pathogenic bacterial strains including *E. coli* (CICC 10389), *S. aureus* (CICC 21600), and *Salmonella enterica* (*S. enterica*, CICC 21482) were grown in Luria-Bertani (LB; Aobox, Beijing, China) broth at 37 °C until mid-logarithmic phase before use. Bacterial suspensions were evenly distributed on LB agar plates and cultured at 37 °C overnight for antimicrobial activity assays.

### DNA extraction

The genomic DNA of isolated LAB strains was extracted using an Invitrogen PureLink^®^ Genomic DNA kit according to the manufacturer’s instructions. The DNA quantity and quality were assessed by the NanoDrop ND-1000 Spectrophotometer (Thermo Fisher Scientific). The DNA was further purified using the Quick-DNA Miniprep Plus kit.

### Library construction and sequencing

For paired-end next-generation sequencing, at least 200 ng genomic DNA was used for sequencing library construction. Paired-end libraries with insert sizes of ~ 400 bp were prepared following standard genomic DNA library preparation procedure. Purified genomic DNA was sheared into fragments with the desired size by Covaris, and blunt ends were generated using T4 DNA polymerase. After adding an ‘A’ base to the 3’ end of the blunt phosphorylated DNA fragments, adapters were ligated to the ends of the DNA fragments. The desired fragments were purified through gel-electrophoresis, then selectively enriched and amplified by PCR. Index tags were introduced via PCR amplification using indexed primers. Library quality and quantity were assessed prior to sequencing. The libraries were finally sequenced on the DNBSEQ-T7 platform (Shanghai BIOZERON Biotech. Co., Ltd) in PE150 mode according to standard protocols.

For third-generation whole-genome sequencing, DNA was pooled into a single multiplexed library and size was selected using Sage Sciences’ BluePippin, which used the 0.75% DF Marker S1 High-Pass 6 kb–10 kb v3 run protocol and S1 marker. A size selection cutoff of 8000 bp (BPstart value) was used. DNA fragments were then purified, end-repaired and ligated with MGI Cyclone sequencing adapters following the manufacturer’s recommendations. Sequencing was performed on the MGI Cyclone platform (Shanghai BIOZERON Biotech. Co., Ltd).

### Genome assembly

Genome assembly was performed using Unicycler (https://github.com/rrwick/Unicycler) [12]. GC depth and genome size information were calculated by custom Perl scripts to assess potential sample contamination. Finally, the strain genome was circularized with Circlator (http://sanger-pathogens.github.io/circlator/).

### Genome annotation

Genome annotation was performed using multiple databases, including non-redundant (NR in NCBI) database, SwissProt (http://uniprot.org), KEGG (http://www.genome.jp/kegg/), GO (http://www.geneontology.org/), COG (http://www.ncbi.nlm.nih.gov/COG), CAZy (http://www.cazy.org/), CARD (https://card.mcmaster.ca/) (e-value ≤ 1e-5, identity ≥ 80%), PHI (e-value ≤ 1e-5), TCDB (http://www.tcdb.org/), VFDB (e-value ≤ 1e-5, identity ≥ 80%), BacMet, SignalP (http://www.cbs.dtu.dk/services/SignalP/), and TMHMM (http://www.cbs.dtu.dk/services/TMHMM/). In addition, tRNAs were identified using the tRNAscan-SE (v2.0.4, http://lowelab.ucsc.edu/tRNAscan-SE) [13] and rRNAs were determined using the RNAmmer (v1.2, http://www.cbs.dtu.dk/services/RNAmmer/) [14]. BAGEL5 (http://bagel5.molgenrug.nl/) and antiSMASH analysis (https://antismash.secondarymetabolites.org/) were employed to identify gene clusters of bacteriocin biosynthesis and secondary metabolite biosynthesis, respectively.

### Phylogenetic tree analysis

Phylogenetic analysis of strains CLPX16 and CLPX19 was performed using the Type Strain Genome Server (TYGS) based on whole-genome similarity-derived intergenomic distances. The genome sequences of CLPX16 and CLPX19 were uploaded to the TYGS platform and compared with available type strain genomes in the reference database. Intergenomic distances were calculated automatically from whole-genome comparisons, and a phylogenetic tree was constructed based on the resulting distance matrix. This genome-based phylogenetic analysis was used to determine the taxonomic affiliations of CLPX16 and CLPX19 at the species level.

### The growth of LAB and acid production

LAB strains were incubated in MRS broth at 37 °C and the OD_600_ absorbance of LAB suspension was measured at 0, 0.5, 1, 1.5, 2, 2.5, 3.0, 3.5, 4, 4.5, 5, 5.5, 6, 8, 10, 12, 14, 16, 18, 20, 22, and 24 h of incubation. In addition, the pH value was determined at 0, 6, 12, 18, 24, 30, and 36 h as previously reported [15]. Each measurement was performed in triplicate.

### Tolerance to acid and bile salts

For the determination of tolerance to acid [16], LAB was incubated in MRS broth at different pH (2, 3, 4, and 6.2) at 37 °C. After 4 h incubation, the OD_600_ absorbance of LAB suspension was measured. The survival of LAB at pH 6.2 was defined as control. The calculation formula of LAB survival rate (%) was as follows:

$$\text{Lab survival rate}\;(\%)=\frac{ApHsample}{ApH6.2}\times\:100\%$$

For the determination of tolerance to bile salts [16], LAB was incubated in MRS broth with or without different concentrations of bovine bile (0.2%, 0.3%, and 0.4%) at 37 °C. After 4 h incubation, the OD_600_ absorbance of LAB suspension was measured. The survival of LAB without bovine bile was defined as control. The calculation formula of LAB survival rate (%) was as follows:

$$\text{Lab survival rate}\;({\%})=\frac{Asample}{Acontrol}\times\:100\%$$

### Antioxidant property of LAB

The DPPH free radical scavenging capacity of LAB was determined using cell-free supernatant (CFS) and cell extracts of LAB according to a previous study [17]. LAB suspension was centrifuged at 7,150×*g* for 5 min to obtain bacterial precipitate. After washing with PBS three times, bacterial precipitate was resuspended in PBS and subjected to ultrasonication on ice (300 W output, 2 s pulse on, 3 s pulse off for a total duration of 10 min). The lysate was centrifuged at 7,150×*g* for 3 min at 4 °C. Finally, the supernatant was collected as cell extracts of LAB. Next, 2 mL of CFS or cell extracts were mixed with 2 mL of DPPH ethanol working solution (0.2 mmol/L) and the mixture was incubated at room temperature in the dark for 30 min. Then, the absorbance was measured at 517 nm. The calculation formula of DPPH scavenging activity (%) was as follows: DPPH scavenging activity (%)$\:=\frac{Acontrol-Asample}{Acontrol}\times\:100\%$. PBS was mixed with DPPH ethanol working solution as control (*A*_*control*_).

### Nitrite degradation capacity of LAB

LAB was incubated in MRS broth containing 200 mg/L of sodium nitrite at 37 °C. After 4, 8, 12, 16, 20, and 24 h of incubation, CFS was collected to determine the concentration of nitrite according to the methods previously reported [15]. The calculation formula of nitrite degradation rate (%) was as follows: nitrite degradation rate (%)$\:=\frac{C0-Csample}{C0}\times\:100\%$. The initial concentration of sodium nitrite was defined as *C*_*0*_.

### Auto-aggregation and co-aggregation

After culturing in the MRS broth as described above, LAB (1 × 10^9^ CFU/mL) suspension was prepared in PBS and incubated at 37 °C for 0, 2, 4, 6, 8, and 24 h. Then, the upper solution was aspirated and the absorbance was measured at 600 nm. The calculation formula of auto-aggregation rate (%) was as follows [16]: auto-aggregation rate (%)$\:=\frac{A0-Asample}{A0}\times\:100\%$. The absorbance of the upper solution at 0 h was defined as *A*_0_.

For the co-aggregation assay [16], LAB (1 × 10^9^ CFU/mL) suspension was mixed with an equal volume of pathogenic bacterial (*E. coli*, *S. aureus*, and *S. enterica*) suspension and incubated at 37 °C for 0 and 2 h. After mixing, the upper solution was aspirated and the absorbance was measured at 600 nm. The calculation formula of co-aggregation rate (%) was as follows: co-aggregation rate (%)$\:=\frac{1/2(A1+A2)-Amix}{1/2(A1+A2)}\times\:100\%$. The absorbance of the upper solution of LAB alone and pathogenic bacteria alone at 0 h was defined as *A*_1_ and *A*_*2*_, respectively. The absorbance of the upper solution of LAB mixture at 2 h was defined as *A*_*mix*_.

### Adhesion to intestinal epithelial cells

Porcine intestinal epithelial IPEC-J2 cells were cultured in DMEM containing 10% FBS at 37 °C with 5% CO_2_ until 90% confluence. Then, cells were digested and seeded into 12-well plates at 1 × 10^5^ cells/well for overnight culture. Next, LAB suspension (5 × 10^6^ CFU) prepared in DMEM containing 10% FBS was added to the cells, followed by incubation for 2 h at 37 °C with 5% CO_2_. After incubation, cells were washed with PBS and then lysed with 0.1% Triton X-100 for 5 min. Adherent LAB were counted on MRS plates as *N*_*adhesion*_ and the adhesion rate was as follows [16]: adhesion rate (%)$\:=\frac{Nadhesion}{N0}\times\:100\%$. The initial number of LAB was defined as *N*_*0*_.

### Antimicrobial activity assay

LAB was cultured in MRS broth at 37 °C for 24 h and then centrifuged at 7,150×*g* for 5 min to collect the CFS. The antimicrobial of CFS was determined by the methods as previously described [16]. Pathogenic bacteria were cultured in LB broth at 37 °C for 20 h and adjusted to 1 × 10^7^ CFU/mL. Next, pathogenic bacterial suspensions were poured into LB agar plates. Wells were punched using Oxford cups, and 100 µL of CFS or MRS broth (as control) was added into each well. After incubation at 37 °C for 24 h, the inhibition zone diameter was measured to determine the antimicrobial activity of LAB.

### Hemolysis assay

LAB was cultured in MRS agar plates supplemented with 5% sheep blood and *S. aureus* was used as a positive control [16]. After incubation at 37 °C for 48 h, hemolysis around colonies was observed and imaged.

### Antibiotic susceptibility assay

The antibiotic susceptibility of LAB was evaluated to determine minimum inhibitory concentration (MIC) values of antibiotics against LAB using broth microdilution assay [18]. LAB suspension (1 × 10^6^ CFU/mL) was first prepared in MRS broth. Then, 50 µL of antibiotics ranging from 0 to 512 µg/mL were added to 96-well plates and mixed with 50 µL of LAB. After 18 h incubation at 37 °C, the MIC was defined as the lowest concentration of antibiotics in the well where there was no visible bacterial growth.

### Statistical analysis

GraphPad Prism 9.0 software (San Diego, CA) was used for statistical analysis. All data are presented as mean ± standard deviation (SD).

## Results and discussion

### Genomic analysis of CLPX16 and CLPX19

**Fig. 1:**
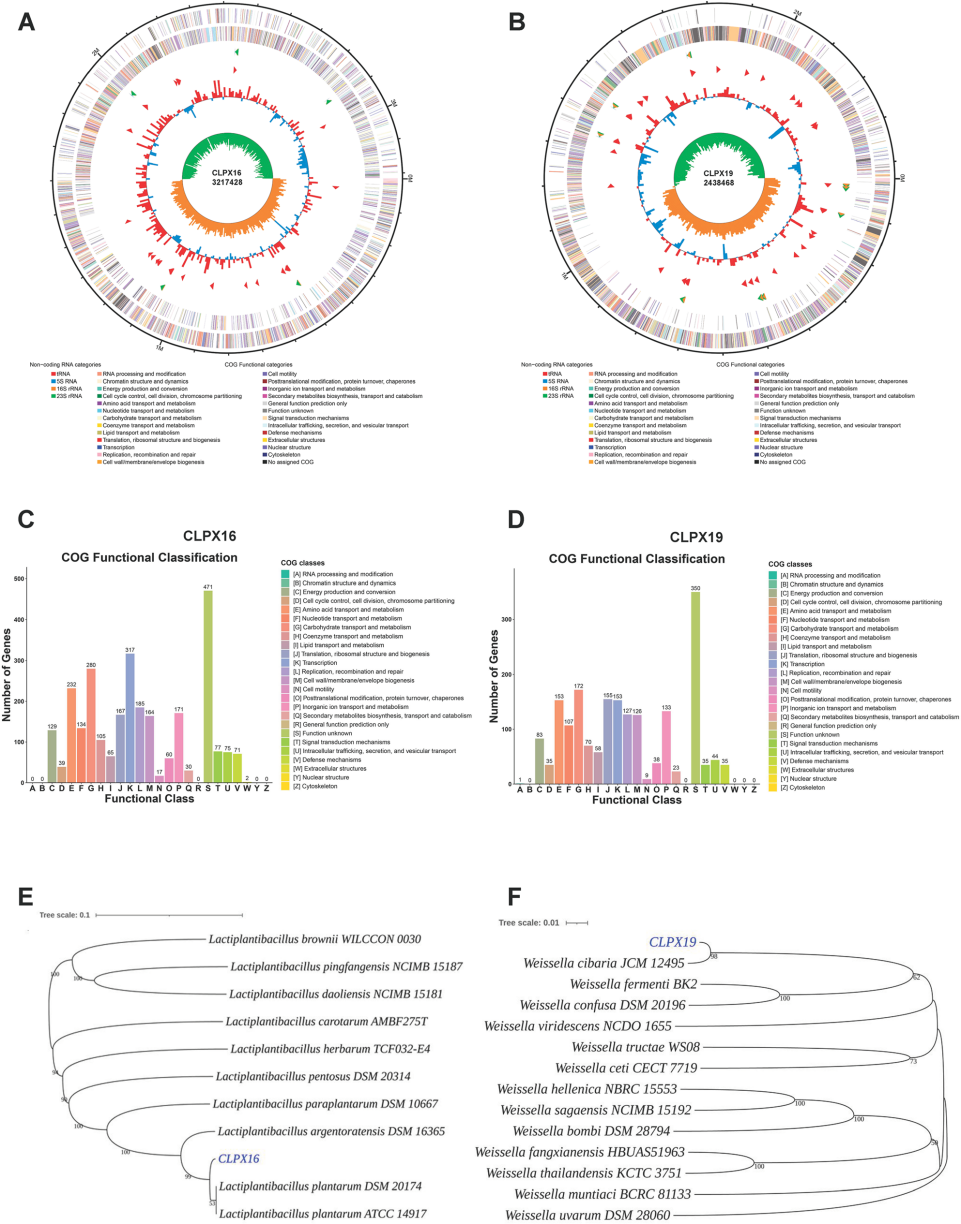
Genomic properties and functional annotation of CLPX16 and CLPX19. **A** Circular genome map of CLPX16. **B** Circular genome map of CLPX19. (**C**, **D**) Distribution of functional categories of annotated genes in the COG database for CLPX16 (**C**) and CLPX19 (**D**). **E**, **F** Phylogenetic analysis of CLPX16 (**E**) and CLPX19 (**F**)

Genome sequencing showed that the CLPX16 genome comprises a 3,217,428-bp chromosome with 44.38% guanine-cytosine (GC) content, and six plasmids with the sizes of 60,054-bp, 58,477-bp, 11,341-bp, 9,976-bp, 7,340-bp, and 1,814-bp as well as 3250 CDSs (Fig. 1A; Table 1). The CLPX19 genome comprises a 2,438,468-bp chromosome with 45.02% GC content and two plasmids with the sizes of 21,805-bp and 3,356-bp as well as 2332 CDSs (Fig. 1B; Table 1). Furthermore, ncRNA in the CLPX16 genome included six 5 S rRNA, five 16 S rRNA, five 23 S rRNA, and 70 tRNA genes. ncRNA in CLPX19 genome included ten 5 S rRNA, nine 16 S rRNA, nine 23 S rRNA, and 88 tRNA genes (Table 1). In addition, the CLPX16 chromosome was predicted to contain one CRISPR gene and nine prophages (Table 1). The CLPX19 chromosome was predicted to contain one CRISPR gene and 14 prophages (Table 1).

**Table 1:** General genome information of CLPX16 and CLPX19 strains

Indicator	Number or content (CLPX16)	Number or content (CLPX19)
Chromosome	3,217,428-bp	2,438,468-bp
Plasmid	6	2
GC content of chromosome	44.38%	45.02%
Coding genes number	3250	2332
Total length of coding genes	2,842,773-bp	2,144,151-bp
23 S rRNA	5	9
16 S rRNA	5	9
5 S rRNA	6	10
tRNA	70	88
CRISPR	1	1
Prophage	9	14

**Fig. 2:**
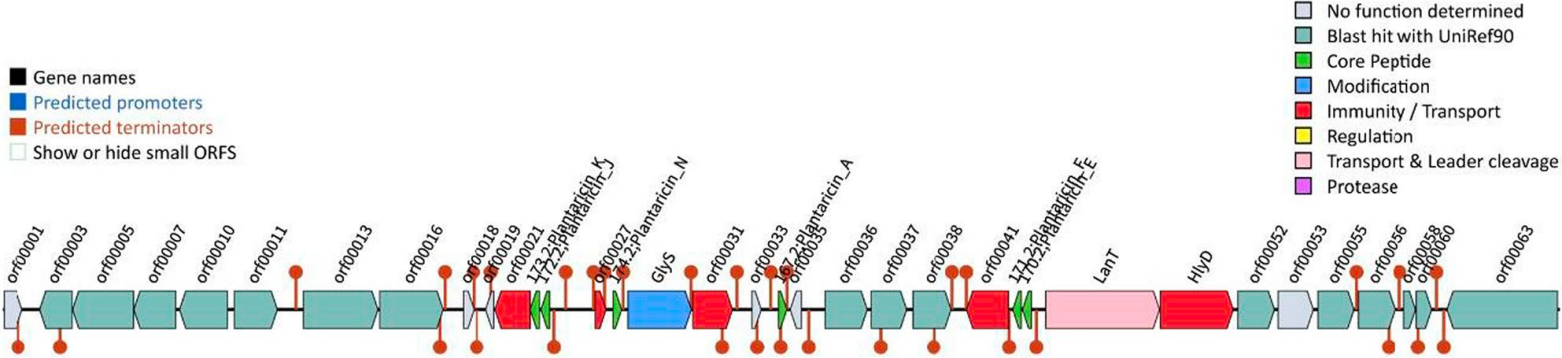
Predicted bacteriocin gene cluster organization in CLPX16. The schematic representation shows the arrangement of ORFs within the cluster, with predicted promoters, terminators, and functional modules indicated by different colors. Core peptides, modification enzymes, immunity proteins, regulatory elements, and transport-related genes are highlighted

COG functional classification showed that genes in both CLPX16 and CLPX19 were predominantly assigned to categories related to translation and ribosomal structure, carbohydrate and amino acid metabolism, transcription, and DNA replication and repair (Fig. 1C and D). Interestingly, a complete bacteriocin gene cluster of approximately 60 kb was detected in CLPX16, comprising at least 63 open reading frames (ORFs). Functional annotation revealed that the cluster could be categorized into genes encoding core peptides, modification enzymes, immunity/transport proteins, regulatory elements, and secretion-related proteins (Fig. 2). The core region harbors multiple genes showing high similarity to the plantaricin family bacteriocins (e.g., PlnA, PlnJ, PlnK, and PlnN) (Table 2), suggesting that CLPX16 produces a class II peptide bacteriocin. Several genes, including orf00021, orf00027, and orf00031, were annotated as bacteriocin immunity proteins, indicating their role in self-protection (Table 2). Notably, gene clusters associated with secondary metabolite biosynthesis were identified in the genomes of CLPX16 (Table S1) and CLPX19 (Table S2). These gene clusters include RiPP-like, terpene-precursor, T3PKS, terpene, and cyclic-lactone-autoinducer in CLPX16, and T3PKS and terpene-precursor in CLPX19, suggesting that both strains have the capacity to produce compounds for antimicrobial, antioxidant, anti-inflammatory, anticancer, and quorum sensing properties [19–21].

**Table 2:** Functional annotation of predicted bacteriocin-related genes in CLPX16

ORF	Gene Strand	Gene Length (bp)	Function
orf00001	+	354	/
orf00003	-	618	Galactoside O-acetyltransferase
orf00005	-	1155	Quinolone resistance protein NorA
orf00010	-	909	HTH-type transcriptional activator RhaR
orf00011	+	816	Sugar phosphatase YidA
orf00013	+	1410	Branched-chain amino acid transport system carrier protein
orf00016	+	1197	Na(+)/H(+) antiporter
orf00018	+	204	/
orf00019	-	153	/
orf00021	-	669	Putative bacteriocin Immunity protein
orf00022	-	174	ComC; Bacteriocin_IIc; 173.2; Plantaricin_K
orf00023	-	168	172.2; Plantaricin_J
orf00027	+	201	P71462_LACPL Immunity protein PlnM
orf00028	+	168	Bacteriocin_IIc; 174.2; Plantaricin_N
orf00029	+	1200	PlnO
orf00031	+	747	P71468_LACPL PlnI (Immunity protein PlnI, membrane-bound protease CAAX family)
orf00033	+	174	/
orf00034	+	156	Antimicrobial17; Bacteriocin_IIc; 167.2; Plantaricin_A
orf00035	-	213	/
orf00036	+	795	Bacteriocin production related histidine kinase
orf00037	+	669	Response regulator PlnC, activator
orf00038	+	744	Response regulator PlnD
orf00041	-	792	P71468_LACPL PlnI (Immunity protein PlnI, membrane-bound protease CAAX family)
orf00044	-	159	ggmotif; Lactococcin; Bacteriocin_IIc; 171.2; Plantaricin_F
orf00046	-	171	170.2; Plantaricin_E
orf00049	+	2151	Bacteriocin ABC-transporter, ATP-binding and permease protein PlnG
orf00050	+	1377	Accessory factor for ABC-transporter PlnH
orf00052	+	690	PlnS
orf00053	+	669	/
orf00055	+	681	PlnS
orf00056	+	696	PlnS
orf00058	+	228	Uncharacterized protein HI_1250
orf00060	+	294	PlnY
orf00063	-	2091	DNA helicase IV

The results of CLPX16 and CLPX19 in the CARD database showed that both strains exhibited antibiotic-resistant genes (ARGs) but the identity of these ARGs was less than 80% (Table S3 and Table S4). Sequences are considered ARGs only when they share at least 80% identity with known resistance genes [22]. The identity of ARGs including *rpoB*, *rpsE*, and *EF-Tu* ranged from 70% to 80% (Table S3 and Table S4). Notably, annotated ARGs related to antibiotic-resistant mechanisms mainly involving in antibiotic efflux and antibiotic target alteration such as *mdtG* and *fusA*, may enhance LAB’s stress resistance [23]. Annotation results against the VFDB database showed several virulence-associated genes in CLPX16 (*hasC*, *clpP*, *eno*, *lisR*, *tufA*) (Table S5) and CLPX19 (*hasC*, *cpsI*, *lisR*, *tuf*, *rfbB*) (Table S6) with below 80% identity. According to EFSA, genes with at least 80% sequence identity are considered virulence factors [24]. In addition, these genes are primarily associated with essential cellular functions including carbohydrate metabolism, stress tolerance, regulatory responses, adhesion, immune modulation, and surface interactions, which are commonly found in nonpathogenic LAB rather than representing true virulence factors.

The whole-genome phylogenetic analysis using TYGS clearly resolved the taxonomic positions of CLPX16 and CLPX19. CLPX16 clustered within the *Lactiplantibacillus* lineage and formed a subclade with *Lactiplantibacillus plantarum* (*L. plantarum*) DSM 20,174 and *L. plantarum* ATCC 14,917, supporting its assignment to *L. plantarum*. CLPX19 clustered closely with *Weissella cibaria* (*W. cibaria*) JCM 12,495 and was clearly separated from other related *Weissella species*, confirming its identification as *W. cibaria* (Fig. 1E, 1F).

### Functional characterization of CLPX16 and CLPX19

#### The growth of CLPX16 and CLPX19

**Fig. 3:**
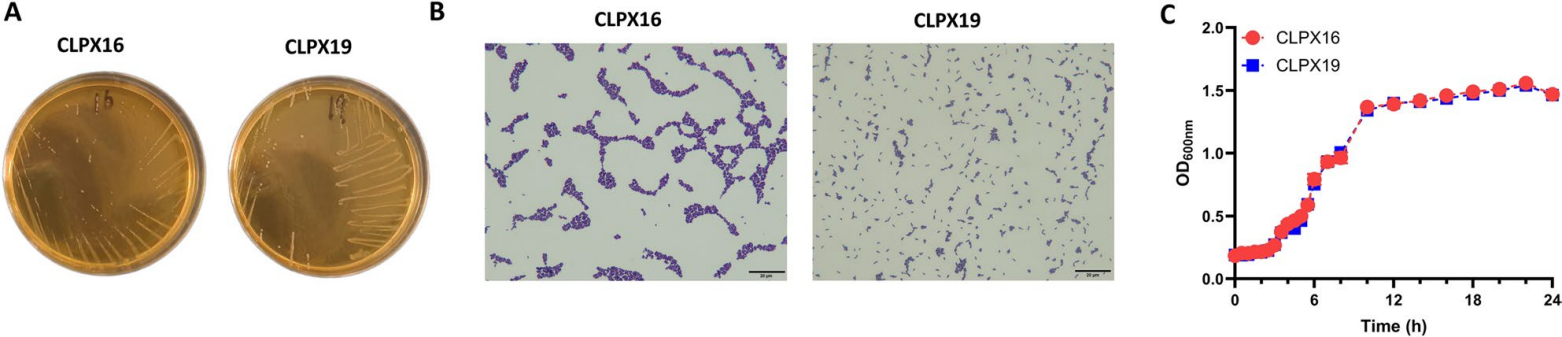
Growth characterization of CLPX16 and CLPX19. **A** Colony morphology of CLPX16 and CLPX19 on MRS agar plates. **B** Morphology by Gram staining. **C** The gowth of CLPX16 and CLPX19

Colony morphology was observed on MRS agar plates. The colonies of CLPX16 and CLPX19 were white and round with smooth surfaces on MRS agar plates (Fig. 3A). Gram staining showed that both strains were Gram-positive with typical rod or coccoid morphology (Fig. 3B). For their growth property, CLPX16 and CLPX19 showed similar growth curves, exhibiting the lag phase, logarithmic phase, and stationary phase (Fig. 3C).

#### Tolerance to acid and bile salts

**Fig. 4:**
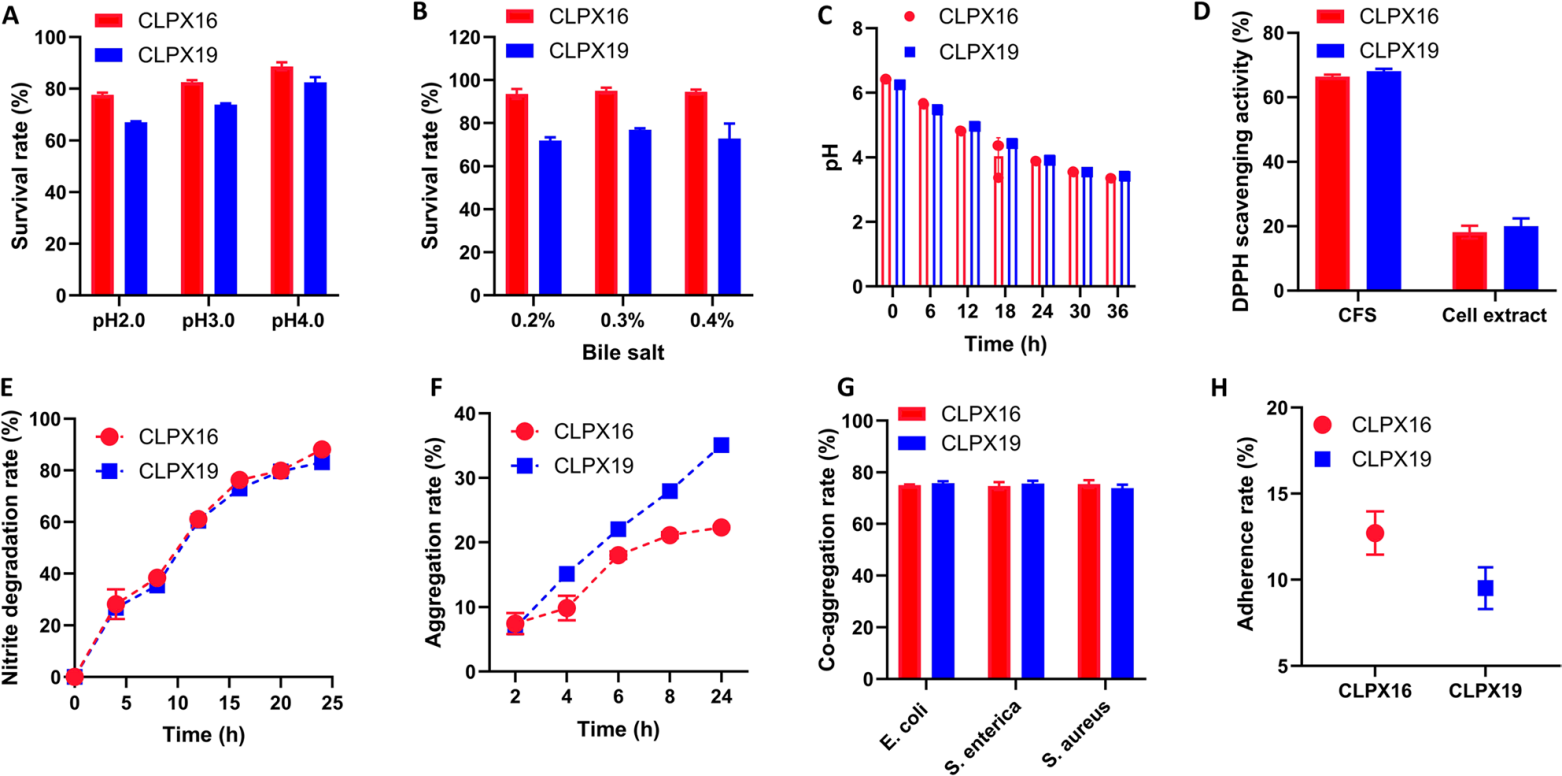
Functional characterization of CLPX16 and CLPX19. **A** Survival rates of CLPX16 and CLPX19 at different pH levels. **B** Survival rates of CLPX16 and CLPX19 at different bile salt concentrations. **C** Acid production-induced pH changes. **D** DPPH free radical scavenging activity. **E** Nitrite degradation rates of CLPX16 and CLPX19. **F** Auto-aggregation rates of CLPX16 and CLPX19. **G** Co-aggregation rates of CLPX16 and CLPX19. **H** Adhesion rates of CLPX16 and CLPX19 towards IPEC-J2 cells

Due to excellent properties including their tolerance to acidic conditions, capacity of adhesion to intestinal mucosa, and bacteriocin production, LAB have been considered to have beneficial effects on the host’s health, especially on the modulation of gut microbiota [11], where LAB face extreme environmental challenges such as low pH and high bile salt concentrations. Therefore, acid and bile salt tolerance have become important properties for the selection of effective LAB. As shown in Fig. 4A, both strains maintained high survival rates under acidic conditions at pH 2.0, 3.0, and 4.0, with survival rates ranging from 77% to 90% for CLPX16 and 67% to 85% for CLPX19. As shown in Fig. 4B, both strains also exhibited good tolerance to bile salts at different concentrations (0.2%, 0.3%, and 0.4%), with survival rates ranging from 91% to 96% for CLPX16 and 67% to 77% for CLPX19. *L. plantarum* MA2 isolated from Tibetan Kefir Grains could survive at pH 2.5 and 0.3% bile salts [25], and *L. plantarum* GCC_19M1 isolated from fermented milk product showed good tolerance to low pH, 0.3% bile, 0.5% pancreatin, and 5% NaCl [26]. When exposed to simulated gastric juice (pH 3.0) for 3 h, the latter strain showed an above 93% survival rate. In our study, both CLPX16 and CLPX19 exhibited strong tolerance to extreme environments, which is consistent with the whole-genome analysis results. Although virulence-related genes were annotated in both strains, genes such as *clpP* [27] in CLPX16 and *lisR* [28] in both strains are associated with stress survival and environmental stress response. These genes might contribute to the probiotic properties, but the exact functional role needs to be further studied.

#### Acid production, antioxidant, and nitrite degradation capacity

Apart from survival in the extreme environment, the formation of organic acids is also an important property of LAB. LAB strains isolated from Chinese pickles, fermented blueberry, or fermented corn peptide were characterized to produce high acid contents during fermentation [29–31]. As shown in Fig. 4C, CLPX16 and CLPX19 exhibited similar acid production capacity, inducing a rapid decline in pH during 0–36 h incubation and reaching the lowest values of 3.36 and 3.42 after 36 h for CLPX16 and CLPX19, respectively. Genomic analysis revealed the presence of Na(+)/H(+) antiporter in the genome of CLPX16 (Table 2), which may regulate intracellular pH in response to acid environments [32].

LAB produce bioactive substances to improve the antioxidant properties of fermented foods. The antioxidant capacity has emerged as a crucial indicator for evaluating LAB with probiotic properties. Hu et al. reported that *L. plantarum* ZNY-04 exhibited excellent DPPH radical scavenging rate (around 80%) [15], and CFS of LAB showed higher antioxidant capacity than cell extracts. Our study showed similar findings. The DPPH free radical scavenging rates of CFS of CLPX16 and CLPX19 were 66.41% and 68.10%, respectively (Fig. 4D). However, the intact cell extracts of CLPX16 and CLPX19 showed significantly lower DPPH radical scavenging rates, which were 18.18% and 20.03%, respectively, suggesting that the antioxidant compounds are predominantly secreted into the extracellular environment rather than retained intracellularly. Consistently, secondary metabolite biosynthesis gene clusters were detected, such as terpene-precursor and terpene, which might contribute to their antioxidative properties [19].

During food fermentation, LAB can reduce nitrite accumulation. *L. plantarum* PK25 utilized carbon source to enhance glycolysis, leading to the formation of lactic acid and acetic acid, which may contribute to nitrite degradation [33, 34]. LAB isolated from kimchi were reported to exert nitrite degradation through directly degrading N-nitrosodimethylamine [35]. In our study, both CLPX16 and CLPX19 exhibited strong nitrite degradation capacity. As shown in Fig. 4E, the nitrite degradation rate increased progressively with incubation time, reaching 88.15% and 83.16% at 24 h for CLPX16 and CLPX19, respectively. This degradation capacity might be attributed to the acidic conditions generated by fermentation, as low pH facilitates nitrite decomposition.

#### Adhesion capacity

LAB adhesion is one of the most important selection properties for probiotics development, which is essential for LAB to colonize in the intestinal tract for regulating gut microbiota balance [4]. Moreover, LAB adhesion activity inhibits the adhesion and colonization of pathogenic bacteria to enhance the host intestinal barrier function [4]. Some adhesion-associated genes such as *tuf* and *tufA* have been predicted and annotated in CLPX16 and CLPX19, which is consistent with the results of in vitro functional assays. The auto-aggregation rate of CLPX16 and CLPX19 increased over time, reaching 22% and 35% at 24 h, respectively (Fig. 4F), which is lower than that reported in other studies [16, 17]. This low auto-aggregation may result from different strains and sources. However, CLPX16 and CLPX19 exhibited strong co-aggregation ability with pathogenic bacteria including *S. aureus*, *E. coli*, and *S. enterica*, with co-aggregation rates reaching approximately 75% (Fig. 4G). Additionally, the adhesion rate of CLPX16 and CLPX19 towards IPEC-J2 cells was 12.71% and 9.51%, respectively (Fig. 4H). The adhesion capacity of CLPX16 and CLPX19 satisfies the key criteria for probiotics, and is comparable to those of other intestinal-derived LAB reported in previous studies [16, 17], indicating their promising application potential in the host.

#### Antimicrobial activity

**Fig. 5:**
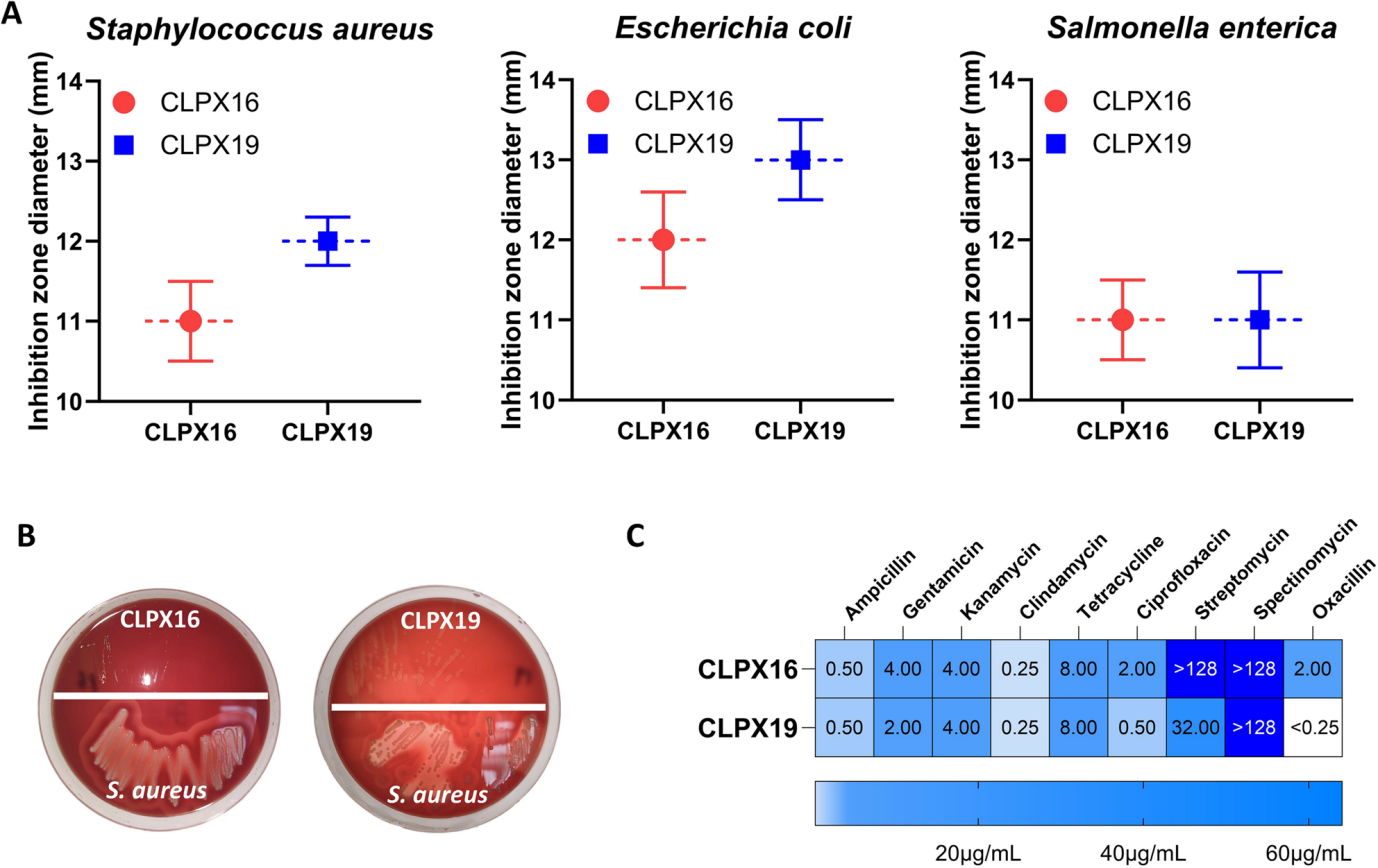
Antimicrobial activity and safety evaluation of CLPX16 and CLPX19 in vitro. **A** Inhibitory effect against *S. aureus*, *E. coli*, and *S. enterica*. **B** Hemolytic activity. **C** Antibiotic susceptibility profiles

Many strains of LAB have been shown to produce antimicrobial compounds, such as short chain fatty acids, bacteriocins, and hydrogen peroxide, which are effective against foodborne pathogens and food spoilage bacteria. *L. plantarum* and *W. cibaria* from fermented foods have exhibited the inhibitory effect on different pathogens, including *E. coli*, *Listeria monocytogenes*, *Salmonella choleraesuis*, *Yersinia enterocolitica*, *Pseudomonas aeruginosa*, *Neisseria gonorrhoeae*,* Klebsiella pneumoniae*,* S. aureus*,* Enterococcus faecium*, *Gardnerella vaginalis*, and *Acinetobacter baumannii* [36–42]. CLPX16 and CLPX19 exhibited substantial inhibitory effects against *S. aureus*,* E. coli*, and *S. enterica* (Fig. 5A). The inhibition zone diameter of CLPX16 against *E. coli*, *S. aureus*, and *S. enterica* was 12, 11, and 11 mm, respectively. The inhibition zone diameter of CLPX19 against *E. coli*, *S. aureus*, and *S. enterica* was 13, 12, and 11 mm, respectively (Fig. 5A). Notably, bacteriocins referred to as plantaricin produced by *L. plantarum* have been characterized as antimicrobial agents and bio-preservatives in the food industry [43]. Various plantaricins from different *L. plantarum* strains have been identified, such as plantaricin MG, plantaricin ZJ5, bacteriocins ST28MS and ST26MS, bacteriocin BacTN635 and bacteriocin Lac-B23, which have shown antimicrobial activity against different pathogens [44–48]. Consistently, gene clusters associated with plantaricin-like bacteriocins were detected in the CLPX16 genome, and secondary metabolite biosynthesis gene clusters were identified in both CLPX16 and CLPX19 genomes, which might contribute to their antimicrobial activities. However, the specific bacteriocins and secondary metabolites responsible for the observed antimicrobial effects need to be further identified.

#### Safety evaluation of CLPX16 and CLPX19 in vitro

Safety evaluation of candidate LAB strains is a critical prerequisite for their application and development. Assessment of hemolytic activity and antibiotic resistance profiles in vitro helps avoid hemolytic damage and horizontal transfer of drug resistance genes, which is essential to exclude potential virulence traits and ensure the biosafety of probiotic strains for host use. Neither CLPX16 nor CLPX19 exhibit hemolytic activity while the positive control *S. aureus* clearly displayed hemolytic activity (Fig. 5B). Notably, both strains exhibited high MIC values for streptomycin and spectinomycin, consistent with the annotation of ARG *rpsE* associated with spectinomycin resistance in the genomic analysis. The streptomycin resistance observed may reflect additional intrinsic mechanisms that warrant further investigation. On the other hand, both strains were susceptible to ampicillin, gentamicin, kanamycin, clindamycin, tetracycline, ciprofloxacin, and oxacillin (Fig. 5C). These results indicate that CLPX16 and CLPX19 display acceptable safety characteristics with no virulence-related risks, which are promising for potential probiotic applications.

## Conclusions

In summary, genomic analysis revealed that both CLPX16 and CLPX19 lack classical virulence factors while harboring beneficial genes, including bacteriocin biosynthesis gene clusters and secondary metabolite gene clusters associated with antimicrobial and antioxidant properties. Functionally, both strains demonstrated strong tolerance to acidic conditions and bile salts, ensuring their survival in the gastrointestinal environment. Furthermore, both strains exhibited multiple beneficial properties including robust acid production capacity, antioxidant activity, nitrite degradation ability, adhesion capacity, and antimicrobial activity against several foodborne pathogens. These genomic and functional characteristics indicate that CLPX16 and CLPX19 have antimicrobial and probiotic potential.

## Supplementary Information

Supplementary Material 1: Table S1. Predicted secondary metabolite biosynthesis gene clusters in CLPX16. Table S2. Predicted secondary metabolite biosynthesis gene clusters in CLPX19. Table S3. Predicted antibiotic resistant genes identified in the genome of CLPX16. Table S4. Predicted antibiotic resistant genes identified in the genome of CLPX19. Table S5. Predicted virulence-associated genes identified in the genome of CLPX16. Table S6. Predicted virulence-associated genes identified in the genome of CLPX19.

## Data Availability

All data are available within the article and from the corresponding author upon reasonable request. The data of genomic analysis have been deposited in the NCBI database under accession number SRR37813416 for CLPX16 and SRR37824834 for CLPX19.
