# Supplementary material for: Whole-genome sequencing and in vitro characterization of two lactic acid bacteria isolates with potent antimicrobial activity from fermented vegetables
Source: BMC Microbiol. 2026 May 18;26:616. doi: 10.1186/s12866-026-05132-2 (PMC13352810; doi:10.1186/s12866-026-05132-2)
Supplement: Supplementary file 1 — Supplementary Material 1: Table S1. Predicted secondary metabolite biosynthesis gene clusters in CLPX16. Table S2. Predicted secondary metabolite biosynthesis gene clusters in CLPX19. Table S3. Predicted antibiotic resistant genes identified in the genome of CLPX16. Table S4. Predicted antibiotic resistant genes identified in the genome of CLPX19. Table S5. Predicted virulence-associated genes identified in the genome of CLPX16. Table S6. Predicted virulence-associated genes identified in the genome of CLPX19. [file 12866_2026_5132_MOESM1_ESM.docx]

**Table S1. Predicted secondary metabolite biosynthesis gene clusters in CLPX16**

| **Region** | **Type** | **From** | **To** |
| --- | --- | --- | --- |
| Region 1 | RiPP-like | 374,564 | 386,714 |
| Region 2 | terpene-precursor | 1,392,332 | 1,413,222 |
| Region 3 | T3PKS | 1,804,913 | 1,846,082 |
| Region 4 | terpene | 2,828,280 | 2,849,161 |
| Region 5 | cyclic-lactone-autoinducer | 3,101,020 | 3,121,725 |

**Table S2. Predicted secondary metabolite biosynthesis gene clusters in CLPX19**

| **Region** | **Type** | **From** | **To** |
| --- | --- | --- | --- |
| Region 1 | T3PKS | 1,471,922 | 1,513,088 |
| Region 2 | terpene-precursor | 1,730,241 | 1,751,104 |

**Table S3. Predicted antibiotic resistance genes identified in the genome of CLPX16**

| **DNA accession** | **Gene name** | **Gene family** | **Identity (%)** | **Species** |
| --- | --- | --- | --- | --- |
| CP000800.1 | mdtG | Phosphonic acid antibiotic | 46.05 | *Escherichia coli* |
| AL009126.3 | rpoB | Rifamycin-resistant | 70.67 | *Bacillus subtilis* |
| BX571856.1 | fusA | Antibiotic resistant | 73.74 | *Staphylococcus aureus* |
| NC_000964.3 | rpsE | Spectinomycin resistant | 71.34 | *Bacillus subtilis* |
| CP003583.1 | EF-Tu | Elfamycin resistant | 75.82 | *Enterococcus faecium* |

**Table S4. Predicted antibiotic resistance genes identified in the genome of CLPX19**

| **DNA accession** | **Gene name** | **Gene family** | **Identity (%)** | **Species** |
| --- | --- | --- | --- | --- |
| CP000800.1 | mdtG | Phosphonic acid antibiotic | 47.75 | *Escherichia coli* |
| CP003583.1 | EF-Tu | Elfamycin resistant | 76.40 | *Enterococcus faecium* |
| NC_000964.3 | rpsE | Spectinomycin resistant | 70.30 | *Bacillus subtilis* |
| AL009126.3 | rpoB | Rifamycin-resistant | 70.67 | *Bacillus subtilis* |

**Table S5. Predicted virulence-associated genes identified in the genome of CLPX16**

| **VFid** | **Function** | **SubType** | **Type** |
| --- | --- | --- | --- |
| VFG005871 | UTP--glucose-1-phosphate uridylyltransferase HasC | hasC | Immune Modulation |
| VFG000077 | ATP-dependent Clp protease proteolytic subunit | clpP | Stress Survival |
| VFG005582 | phosphopyruvate hydratase | eno | Exoenzyme |
| VFG006826 | two-component response regulator | lisR | Regulation |
| VFG046474 | elongation factor Tu | tufA | Adherence |

**Table S6. Predicted virulence-associated genes identified in the genome of CLPX19**

| **VFid** | **Function** | **SubType** | **Type** |
| --- | --- | --- | --- |
| VFG005879 | UTP--glucose-1-phosphate uridylyltransferase HasC | hasC | Immune Modulation |
| VFG002182 | UDP-galactopyranose mutase | cpsI | Immune Modulation |
| VFG006826 | two-component response regulator | lisR | Regulation |
| VFG016490 | elongation factor Tu | tuf | Adherence |
| VFG006022 | dTDP-glucose 4,6-dehydratase | rfbB | Immune Modulation |
